# Accuracy and Validity of Resting Energy Expenditure Predictive Equations in Middle-Aged Adults

**DOI:** 10.3390/nu10111635

**Published:** 2018-11-02

**Authors:** Francisco J. Amaro-Gahete, Lucas Jurado-Fasoli, Alejandro De-la-O, Ángel Gutierrez, Manuel J. Castillo, Jonatan R. Ruiz

**Affiliations:** 1 Department of Medical Physiology, School of Medicine, University of Granada, 18071 Granada, Spain; juradofasoli@ugr.es (L.J.-F.); delao@ugr.es (A.D.-l.-O.); gutierre@ugr.es (Á.G.); mcgarzon@ugr.es (M.J.C.); 2 Promoting Fitness and Health through physical activity research group (PROFITH), Department of Physical Education and Sports, Faculty of Sport Sciences, University of Granada, 18071 Granada, Spain; ruizj@ugr.es

**Keywords:** metabolic rate, basal metabolism, indirect calorimetry, energy balance, obesity

## Abstract

Indirect calorimetry (IC) is considered the reference method to determine the resting energy expenditure (REE), but its use in a clinical context is limited. Alternatively, there is a number of REE predictive equations to estimate the REE. However, it has been shown that the available REE predictive equations could either overestimate or underestimate the REE as measured by IC. Moreover, the role of the weight status in the accuracy and validity of the REE predictive equations requires further attention. Therefore, this study aimed to determine the accuracy and validity of REE predictive equations in normal-weight, overweight, and obese sedentary middle-aged adults. A total of 73 sedentary middle-aged adults (53% women, 40–65 years old) participated in the study. We measured REE by indirect calorimetry, strictly following the standard procedures, and we compared it with the values obtained from 33 predictive equations. The most accurate predictive equations in middle-aged sedentary adults were: (i) the equation of FAO/WHO/UNU in normal-weight individuals (50.0% of prediction accuracy), (ii) the equation of Livingston in overweight individuals (46.9% of prediction accuracy), and (iii) the equation of Owen in individuals with obesity (52.9% of prediction accuracy). Our study shows that the weight status plays an important role in the accuracy and validity of different REE predictive equations in middle-aged adults.

## 1. Introduction

Obesity is associated with an increased morbidity and mortality risk and is considered a significant burden to health care systems worldwide [1]. The number of overweight and obese individuals has globally increased from 857 million to 2.1 billion during the last thirty years, making excess weight a public health problem of the current society [2]. Although the physiological mechanisms that determine or influence obesity are complex, several studies have shown that an energy imbalance between energy intake and energy expenditure is a predisposing factor for metabolic diseases [1].

Total energy expenditure is the sum of resting energy expenditure (REE), physical activity energy expenditure, and thermic effect of food. The REE accounts for more than 50% of the total daily energy expenditure [3]. Indirect calorimetry (IC) is considered the reference method to determine the REE through the determination of O_2_ consumption and CO_2_ production [4]. However, the use of IC in a clinical context is limited because of its strict evaluation conditions, the high cost of the gas analyzer used for its measurement, and the fact that the measurement devices are not usually portable [5]. Alternatively, there is a number REE predictive equations to estimate the REE [6,7,8,9,10,11,12,13,14,15,16,17,18,19,20,21,22,23,24,25]. Previous studies have shown that the available REE predictive equations could either overestimate or underestimate the REE as measured by IC [26,27,28]. Furthermore, the majority of REE predictive equations were proposed decades ago and are based on some specific individual cohorts that had different biological and metabolic characteristics from those of the current population. Moreover, the role of the weight status in the accuracy and validity of the REE predictive equations requires further attention, since individuals with different weight statuses may have different amounts of metabolically active tissues (fat mass versus fat-free mass), which could influence the REE estimation [29].

Therefore, the purpose of this study was to determine the accuracy and validity of REE predictive equations in normal-weight, overweight, and obese sedentary middle-aged adults.

## 2. Materials and Methods

### 2.1. Participants

Seventy-three healthy sedentary adults (53% women), aged between 40 and 65, with a body mass index (BMI) in the range of 20–38 kg/m^2^, Caucasian, non-physically active (<20 min on 3 days/week), and with stable weight (weight changes <5 kg) over the last 6 months participated in the study. The participants were enrolled in the FIT-AGEING study (ClinicalTrials.gov: NCT03334357 (8–11–17)) [30]. We used the baseline data of the original study for data analysis. An informed consent was signed by each participant before they started the intervention program. The study was in accordance with the latest revision of the Helsinki Declaration and it was approved by The Human Research Ethics Committee of the “Junta de Andalucia” (0838-N-2017).

### 2.2. Body Composition

The weight was measured before the REE test using an electronic scale (SECA Alpha 760, Hamburg, Germany) to the nearest 0.1 kg. The height was also measured using a stadiometer (SECA 220, Hamburg, Germany) to the nearest 0.1 cm. The BMI was calculated as weight (kg)/height (m^2^) [31]. The body composition (fat mass, fat-free mass, and lean mass) was determined by Dual-Energy X-ray Absorptiometry (DXA, HOLOGIC, Discovery, Toronto, ON, Canada).

### 2.3. Resting Energy Expenditure Assessment by Indirect Calorimetry

The REE was evaluated by IC following the current recommendations to ensure the validity of the test [32,33]. The participants arrived at the laboratory at 8–9 a.m. after a 12-h fasting period. The participants were asked not to perform any physical activity 48 h before the test. The REE was evaluated in a quiet and relaxing room at a constant temperature (22.6 ± 0.8 °C) and humidity (44.5 ± 6.7%). The participants lay on a bed in a supine position and were asked not to fall asleep. The respiratory exchange was measured after resting for 30 min, using a CPX Ultima CardiO_2_ system (Medical Graphics Corp, St Paul, MN, USA) and a neoprene facemask, equipped with a directconnect™ metabolic flow sensor (Medgraphics Corp, MN, USA). The data were collected during 30 min. The first 5 min of each measurement were routinely discarded, and the most stable 5-min steady state period was selected for the analysis (Breeze Software, MGC Diagnostic^®^, Breeze Suite 8.1.0.54 SP7) [34]. The steady-state criteria were established as: (i) <10% coefficient of variance in VO_2_ consumption, CO_2_ production, and ventilation, and (ii) <5% coefficient of variance in respiratory quotient [35,36]. The REE was calculated from the O_2_ consumed and the CO_2_ produced by using Weir abbreviated equation, assuming that urinary nitrogen excretion was negligible, and it was expressed as kcal/day [37]:

REE = (3.9 (VO_2_) + 1.1 (VCO_2_)) × 1.44. (1)

### 2.4. REE Predictive Equations

The National Library of Medicine’s search service (PUBMED) was used to conduct a systematic search, combining the following keywords: ‘Energy metabolism’, ‘Basal metabolism’, ‘Indirect calorimetry’, and also additional terms (‘rest*‘, ‘measure*‘, ‘predict*’, ‘estimat*’, ‘equation*’, and ‘formula*’).

We only selected the REE predictive equations that complied with the following criteria: (i) developed in adults and (ii) based on weight, height, age, sex, and/or fat mass, fat-free mass, and lean body mass. We excluded the REE predictive equations: (i) conducted in patients with any disease or athlete cohorts, (ii) including a small sample size (*n* < 50), (iii) conducted in specific ethnic groups. A total of 33 predictive equations (see Table S1) were retained and used for the analysis [6,7,8,9,10,11,12,13,14,15,16,17,18,19,20,21,22,23,24].

### 2.5. Statistical Analysis

An analysis of covariance (ANCOVA) was performed to compare measured (by IC) versus predicted REE (by REE predictive equations) adjusting by age and sex. The BIAS (mean error between measured and predicted REE), the absolute differences (measured minus predicted REE in absolute terms), and the 95% limits of agreement were also analyzed. We determined the following two accuracy levels: (i) ±10% of measured REE, which included REE predicted values between 90% and 110% of the measured REE [38,39], considering underprediction when the estimation value was below 90% and overprediction when the estimation value was above 110% of the measured REE, and (ii) ±5% of measured REE, which included REE predicted values between 95% and 105% of the measured REE, considering underprediction when the estimation value was below 95% and overprediction when the estimation value was above 105% of the measured REE.

Repeated measures analysis of variance (ANOVA) across the REE predictive equations was used to determine differences between the REE predictive equation that presented the least absolute differences with respect to the measured REE, respectively.

The heteroscedasticity was tested using the Bland–Altman method [40], which plots the difference between predicted and measured REE versus the mean of predicted and measured REE.

We conducted one-way ANOVA to determine differences across weight status categories (i.e., individuals with normal-weight, overweight, and with obesity) in the percentage of accurate prediction and mean differences between predicted and measured REE in absolute values of the most accurate predictive equations. Although no statistically significant differences in 10 or 5% accurate prediction was found among most of the equations, we selected the most accurate REE predictive equations for each body weight category, based on the percentage of accurate prediction at ±10% of the measured REE. If two or more REE predictive equations provided a similar percentage of accurate prediction at ±10% of the measured REE, we selected the most accurate REE predictive equation at ±5% of the measured REE.

The analyses were conducted using the SPSS version 25.0 (IBM SPSS Statistics, IBM Corporation, Armonk, NY, USA). The analyses were conducted separately in normal-weight, overweight, and obese individuals. The results are expressed as mean ± standard deviation, and the level of statistical significance was set at <0.05.

## 3. Results

**Figure 1:**
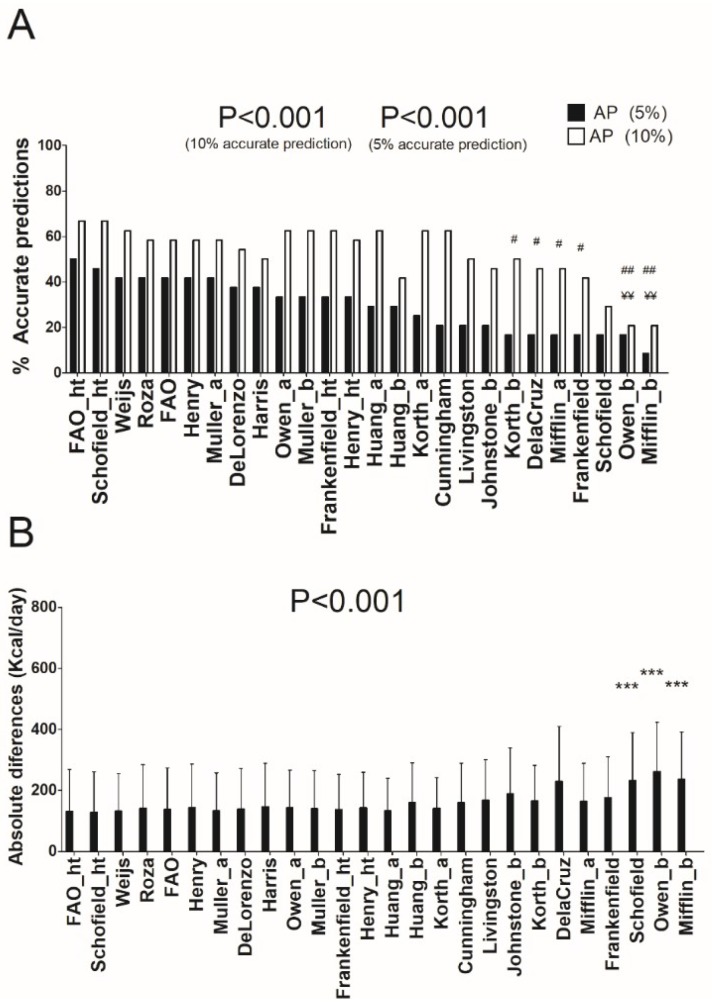
Percentage of accurate prediction of resting energy predictive equations and mean differences between predicted and measured resting energy expenditure in absolute values in normal-weight individuals. (**A**) Percentage of prediction accuracy at 5% and 10% of resting energy expenditure. (**B**) Mean (SD) differences between predicted and measured resting energy expenditure in absolute values; *p* value of repeated measures analysis of variance (with Bonferroni post-hoc analysis) among the predictive equations; * = *p* < 0.05; ** = *p* < 0.01; *** = *p* < 0.001 when compared with the predictive equation that presented the least absolute differences with respect to the measured resting energy expenditure (FAO_ht); ¥ = *p* < 0.05; ¥¥ = *p* < 0.01; ¥¥¥ = *p* < 0.001 when compared with the predictive equation that presented the best resting energy expenditure prediction accuracy (10%) with respect to the measured resting energy expenditure (FAO_ht); # = *p* < 0.05; ## = *p* < 0.01; ### = *p* < 0.001 when compared with the predictive equation that presented the best resting energy expenditure prediction accuracy (5%) with respect to the measured resting energy expenditure (FAO_ht). AP: Accurate prediction; “_a” refers to predictive equations which require only anthropometric parameters to calculate REE, “_b” refers to predictive equations which require body composition parameters to calculate REE, and “_ht” refers to predictive equations which are proposed by the same author and include height.

Table 1 shows the characteristics of the study sample. In normal-weight individuals (see Figure 1A and Supplementary Table S2A), the Schofield [12] and FAO/WHO/UNU [13] predictive equations presented 66.7% of prediction accuracy, 20.8% underpredictions, and 12.5% overpredictions (accurate prediction ±10%). Nevertheless, when a severe accurate estimation (±5%) was applied, the equation of FAO/WHO/UNU [13] provided 50.0% of prediction accuracy, and the equation of Schofield [12] 45.8% of prediction accuracy (mean absolute differences: 131 ± 138 and 129 ± 132 Kcal/day, respectively). Repeated measures ANOVA showed significant differences (all with *p* < 0.001) when comparing the REE estimation by the equation of FAO [13] with those by the equations of Owen [8,9] and Mifflin [10] (see Figure 1B). The results persisted when including age and sex as covariates (all *p* > 0.3).

**Figure 2:**
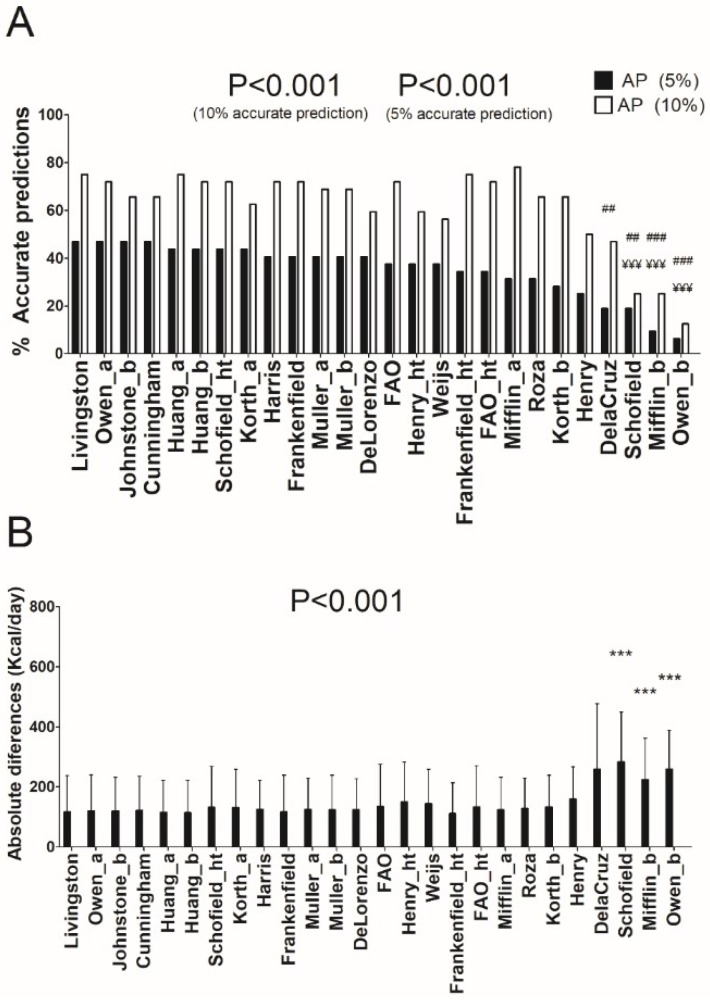
Percentage of accurate prediction of resting energy predictive equations and mean differences between predicted and measured resting energy expenditure in absolute values in overweight individuals. (**A**) Percentage of prediction accuracy at 5% and 10% of resting energy expenditure. (**B**) Mean (SD) differences between predicted and measured resting energy expenditure in absolute values; *p* value of repeated measures analysis of variance (with Bonferroni post-hoc analysis) among the predictive equations; * = *p* < 0.05; ** = *p* < 0.01; *** = *p* < 0.001 when compared with the predictive equation that presented the least absolute differences with respect to the measured resting energy expenditure (Livingston); ¥ = *p* < 0.05; ¥¥ = *p* < 0.01; ¥¥¥ = *p* < 0.001 when compared with the predictive equation that presented the best resting energy expenditure prediction accuracy (10%) with respect to the measured resting energy expenditure (Roza); # = *p* < 0.05; ## = *p* < 0.01; ### = *p* < 0.001 when compared with the predictive equation that presented the best resting energy expenditure prediction accuracy (5%) with respect to the measured resting energy expenditure (Livingston). AP: Accurate prediction; “_a” refers to predictive equations which require only anthropometric parameters to calculate REE, “_b” refers to predictive equations which require body composition parameters to calculate REE, and “_ht” refers to predictive equations which are proposed by the same author and include height.

Figure 2A,B show the percentage of prediction accuracy in all REE predictive equations and the mean absolute values differences between predicted and measured REE in overweight participants, respectively. The equations of Livingston [11] and Huang [23] provided a similar percentage of prediction accuracy (75%) when ±10% of accurate estimation was applied. However, when a severe accurate estimation filter (±5%) was applied, the equation of Livingston [11] showed the highest percentage of prediction accuracy (46.9% versus 43.8%, respectively). The absolute differences were 117 ± 122 and 114 ± 109 Kcal/day for Livingston´s [11] and Huang´s REE predictive equations [23], respectively (see Supplementary Table S2B). An interaction effect in ANCOVA analysis was observed adjusting by age in the equations of Schofield [12] (*p* = 0.003) and Owen [8,9] (*p* = 0.042), whereas no sex interaction was observed in the model (*p* > 0.4). We also noted significant differences (all *p* < 0.01) when we compared the REE estimation (in absolute values) by the equation of Livingston [11] versus those by the equations of Schofield [12], Mifflin [10], and Owen [9] (see Figure 2B).

**Figure 3:**
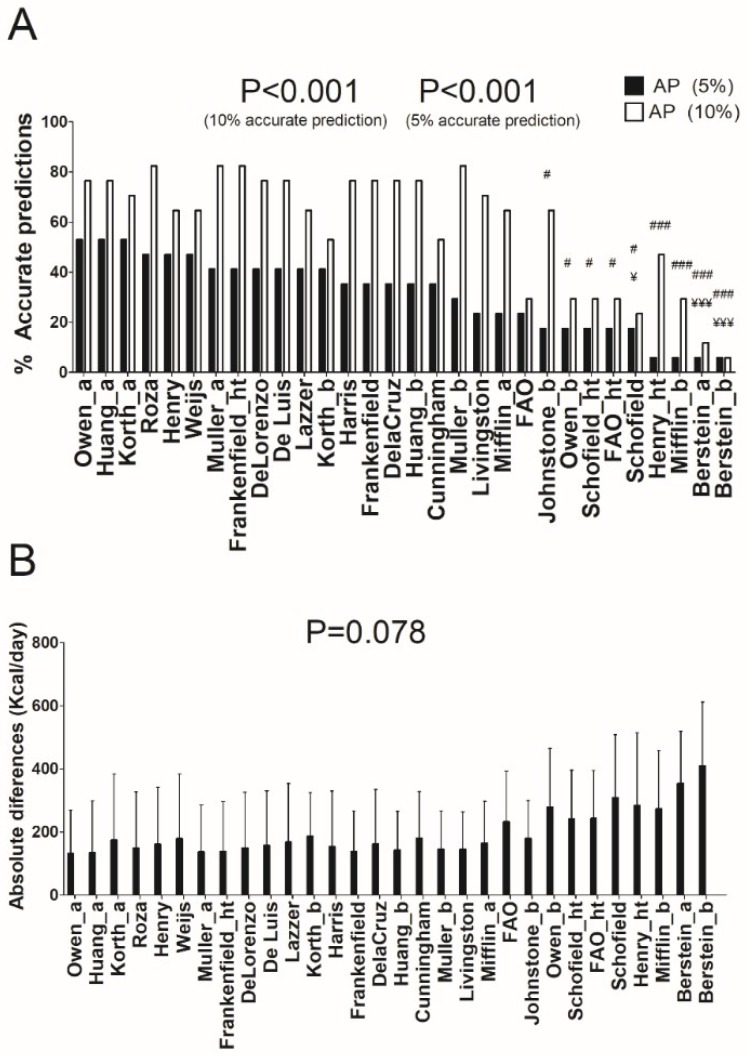
Percentage of accurate prediction of resting energy predictive equations and mean differences between predicted and measured resting energy expenditure in absolute values in individuals with obesity. (**A**) Percentage of prediction accuracy at 5% and 10% of resting energy expenditure. (**B**) Mean (SD) differences between predicted and measured resting energy expenditure in absolute values; *p* value of repeated measures analysis of variance (with Bonferroni post-hoc analysis) among the predictive equations; * = *p* < 0.05; ** = *p* < 0.01; *** = *p* < 0.001 when compared with the predictive equation that presented the least absolute differences with respect to the measured resting energy expenditure (Owen_a); ¥ = *p* < 0.05; ¥¥ = *p* < 0.01; ¥¥¥ = *p* < 0.001 when compared with the predictive equation that presented the best resting energy expenditure prediction accuracy (10%) with respect to the measured resting energy expenditure (Roza); # = *p* < 0.05; ## = *p* < 0.01; ### = *p* < 0.001 when compared with the predictive equation that presented the best resting energy expenditure prediction accuracy (5%) with respect to the measured resting energy expenditure (Owen_a). AP: Accurate prediction; “_a” refers to predictive equations which require only anthropometric parameters to calculate REE, “_b” refers to predictive equations which require body composition parameters to calculate REE, and “_ht” refers to predictive equations which are proposed by the same author and include height.

In individuals with obesity, several REE predictive equations provided 82.4% of prediction accuracy (accurate prediction ±10%, see Figure 3A) [7,15,37], yet, when a severe accurate estimation was applied (±5% of measured REE), the equation of Owen [8,9] showed the highest accuracy (52.9% of prediction accuracy; absolute differences: 132 ± 138 Kcal/day). An interaction effect was observed adjusting by age in De Lorenzo [41] and Lazzer [42] predictive equations (both *p* < 0.05, see Supplementary Table S2C), whereas no interaction was observed adding sex in the model (*p* > 0.2). Repeated measures ANOVA did not show significant differences between all predictive equations in terms of absolute differences (*p* = 0.078) (see Figure 3B).

**Figure 4:**
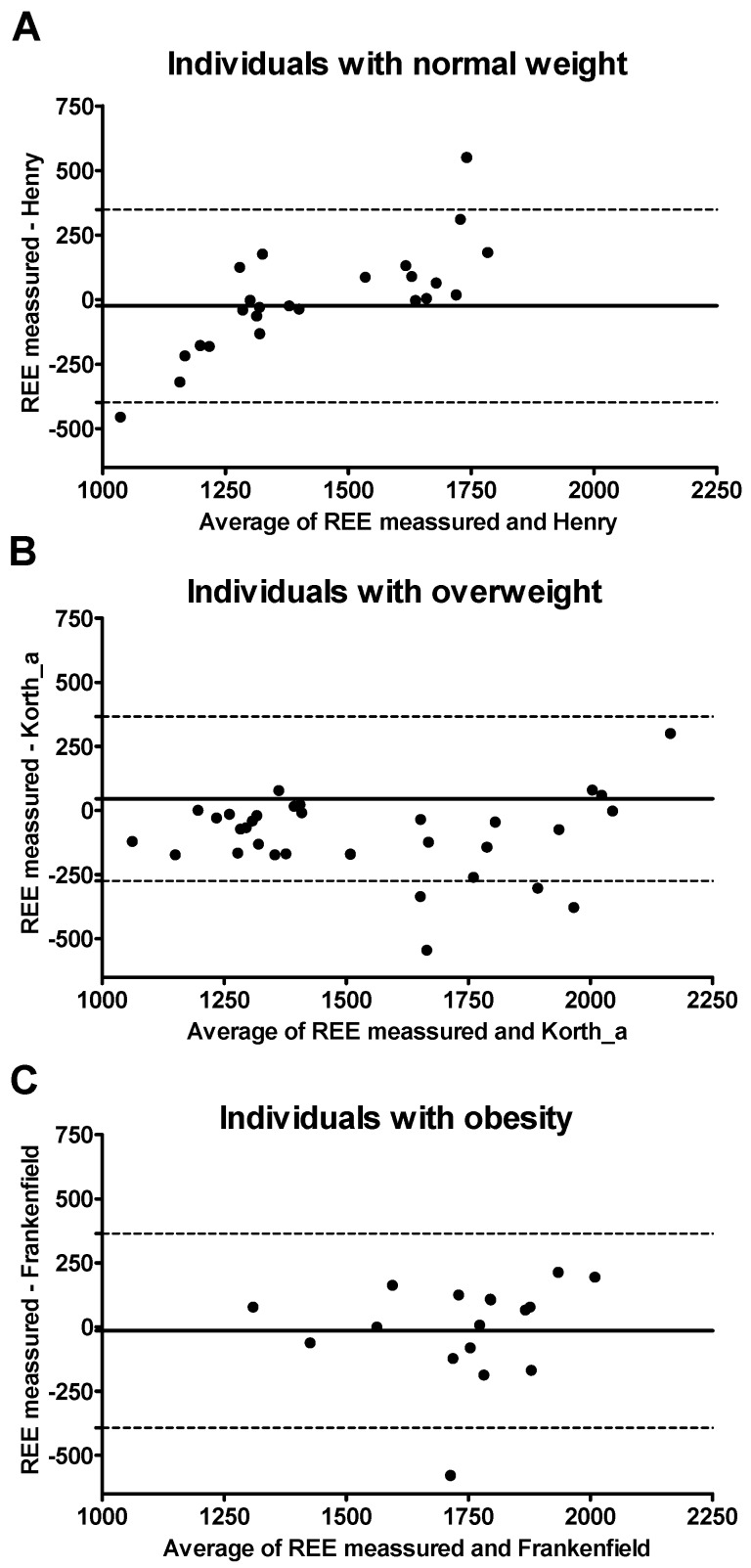
Bland–Altman plots for selected resting energy expenditure (REE) predictive equations. The solid lines represent the mean difference (BIAS) between predicted and measured REE. The upper and lower dashed lines represent the 95% limits of agreement; “_a” refers to predictive equations which required only anthropometric parameters to calculate REE, and “_ht” refers to predictive equations which are proposed by the same author and include height.

Figure 4 shows Bland–Altman plots for the three selected REE predictive equations and measured REE by weight status. The limits of agreement were the following: (i) −496 to 373 Kcal/day in normal-weight participants (using the equation of FAO/WHO/UNU [13], see Figure 4A and Supplementary Table S2A), (ii) −249 to 562 Kcal/day in overweight participants (using the equation of Livingston [11], see Figure 4B and Supplementary Table S2B), and (iii) −591 to 245 Kcal/day in individuals with obesity (using the equation of Owen [8,9,11], see Figure 4C and Supplementary Table S2C). 

**Figure 5:**
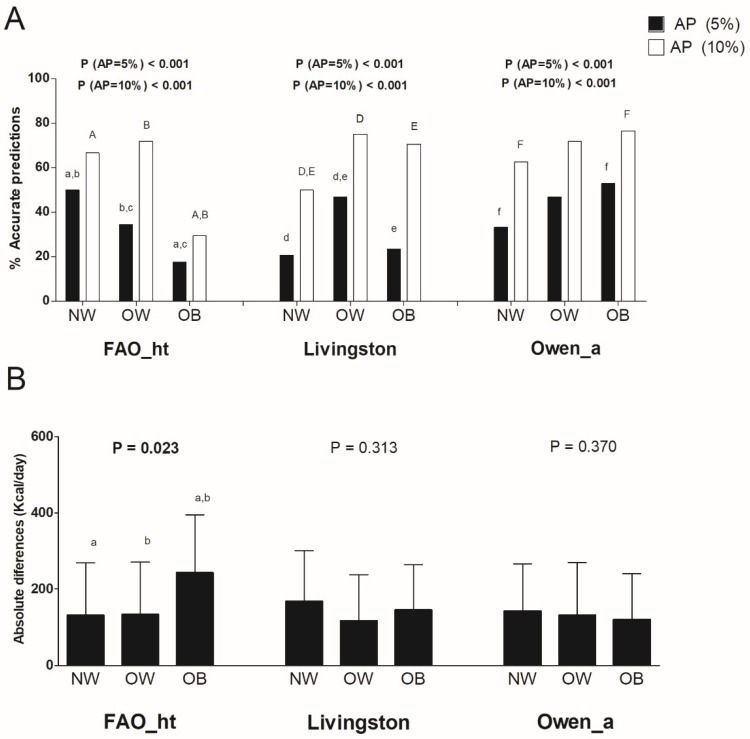
Percentage of accurate prediction of the most accurate predictive equations and mean differences between predicted and measured resting energy expenditure in absolute values by weight status. (**A**) Percentage of prediction accuracy at 5% and 10% of resting energy expenditure. (**B**) Mean (SD) differences between predicted and measured resting energy expenditure in absolute values; *p* value of an analysis of variance (with Bonferroni post-hoc analysis) across weight status. Similar letters (i.e., a-a, b-b) indicate significant differences (*p* < 0.05) considering Bonferroni post-hoc analysis. AP: Accurate prediction; “_a” refers to predictive equations which require only anthropometric parameters to calculate REE, and “_ht” refers to predictive equations which are proposed by the same author and include height.

Figure 5 shows the comparison of the most accurate predictive equations for individuals with normal weight, individuals with overweight, and individuals with obesity, respectively, by weight status. We observed significant differences in the percentage of accurate predictions applying both the ±10% and the ±5% of measured REE criteria in FAO_ht, Livingston, and Owen_a predictive equations (all *p* < 0.001, see Figure 5A). No significant differences were noted comparing the mean differences between predicted and measured resting energy expenditure in absolute values by weight status in Livingston and Owen_a predictive equations (All *p* > 0.313, see Figure 5B), while significant differences were observed considering FAO_ht equation (*p* = 0.023, see Figure 5B).

## 4. Discussion

**Figure 6:**
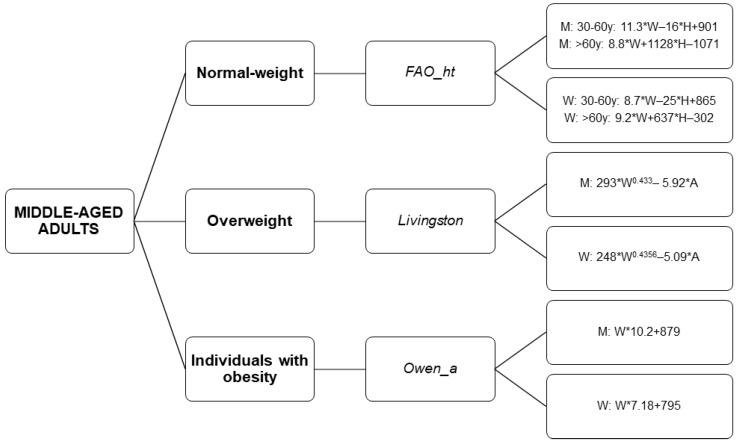
Decision tree to select a REE predictive equation by weight status. “_a” refers to predictive equations which require only anthropometric parameters to calculate REE, and “_ht” refers to predictive equations which are proposed by the same author and include height. Abbreviations: M: Men; W: Women; W: Weight; H: Height; A: Age; y: years.

The present study identifies the most accurate predictive equations by weight status in middle-aged sedentary adults: (i) the equation of FAO/WHO/UNU [13] in normal-weight individuals, (ii) the equation of Livingston [11] in overweight individuals, and (iii) the equation of Owen [8,9] in individuals with obesity. Moreover, there were significant differences in the percentage of accurate prediction when comparing the REE estimated values provided by the most accurate predictive equations for each weight status category. We also provide a flowchart decision tree to choose the REE predictive equation by weight status (see Figure 6), considering (i) the % of prediction accuracy applying an accuracy level of ±5%, and (ii) the % of prediction accuracy applying an accuracy level of ±10%.

Our results suggest that the best equation to estimate REE in normal-weight adults is the equation of FAO/WHO/UNU [13]. Our results differ from those of another study [37] that showed that the equation of Mifflin [10] was the most accurate REE predictive equation (68% of accuracy prediction) when an accuracy level of ±5% was applied. These differences could be explained by the lack of details reported by Frankenfield et al. [37] regarding the IC analysis criteria to determine the REE and the inclusion of a heterogeneous individual population. In a cohort of Belgian normal-weight women, the most accurate REE predictive equation was the Huang equation [43], with 71% of prediction accuracy. Our results also revealed a good prediction accuracy with the equation of Huang (62.5% of prediction accuracy) [43]. However, these differences might be explained by three specific facts: (i) Weijs et al. [43] only considered women, (ii) they selected 20-min steady state periods to obtain the REE measurement (we selected the most stable 5-min steady state period), and (iii) the gas analyzer device was different in both studies.

Our results provide more evidence for the use of the Livingston equation [11] in overweight individuals (46.9% prediction accuracy, mean absolute differences: 116.5 ± 121.9 Kcal/day) and concur with another study performed in normal-weight, overweight, and obese individuals (55% prediction accuracy) [44]. However, a systematic review conducted in overweight individuals [45] reported higher accuracy when the Harris–Benedict predictive Equation (7) was applied (62.7% of prediction accuracy), whereas we obtained 49.9% of prediction accuracy when using the same equation. These facts could be explained by the inclusion of numerous studies with different gas collection systems (e.g., direct calorimetry versus IC), different gas analyzers used to determine REE (e.g., Vmax Encore n29, Viasys Healthcare versus Oxycon Pro, Erich Jaeger GmbH, Hoechberg, Germany, between others), and also different population groups (e.g., overweight U.S. adults versus normal-weight European women).

In individuals with obesity, the equation of Owen [8,9] showed the highest accuracy values (52.9% of prediction accuracy, mean absolute differences: 131 ± 137 Kcal/day), which concur with another study conducted in Australian individuals with obesity (~47 years of age, 41.8% of prediction accuracy at ±10% of accuracy level) [43]. However, a recent systematic review [36] suggested that the equation of Mifflin [10] was the most accurate predictive equation for individuals with obesity (48% of prediction accuracy, applying an accuracy level of ±5%) [37], which differs from our results (23.5% of prediction accuracy). This difference might be partially explained by the inclusion of only five REE predictive equations in Frankenfield et al. [37]: Mifflin [10], Livingston [11], Muller [15], Harris Benedict (16), and FAO/WHO/UNU [13] equations. The Mifflin equation [10] has also been proposed as the most accurate REE predictive equation in Belgian women with obesity [42] (68% of accuracy at ±10% accuracy level), in Taiwanese individuals with obesity (46.3% of accuracy at ±10% accuracy level) [46], and in 1900 Italian individuals with obesity (39.7% of accuracy at ±10% accuracy level).

We noted that the inclusion of body composition parameters (fat mass, fat-free mass, or lean mass) did not improve the accuracy of the REE prediction in our participants. This is especially relevant because age-, weight-, and height-derived equations are more feasible in the clinical practice.

The results of this study should be considered with caution: (i) our participants were middle-aged healthy sedentary adults (45–65 years of age), hence, we cannot extend our results to older or younger individuals, (ii) although we did not find interaction with sex, our results need to be confirmed studying the role of sex and weight status together, (iii) although it is well known that metabolic carts can overestimate or underestimate the REE measure, it is important to consider that our data collection and the analysis process were strictly controlled and standardized, (iv) the respiratory exchange was measured using a neoprene facemask and not a canopy, as it is usually the case, and this issue could influence the results of the validity of the equations used to estimate REE, (v) the small sample size of women with obesity and (vi) the use of the Weir equation implies assumptions that may not be accurate enough (e.g., absence of protein oxidation). Consequently, this could prevent us from extending our results to other populations with obesity, which present higher fat mass values compared to overweight populations.

## 5. Conclusions

In conclusion, our study shows that the REE predictive equation varies depending on the weight status for sedentary middle-aged adults. Future studies must be conducted in order to confirm the results obtained in older and younger individuals. We also provide an open access Excel sheet that automatically estimates the REE and the total energy expenditure using 33 equations considering anthropometric and/or body composition parameters (if available).

## Figures and Tables

**Table 1:** Descriptive parameters.

	All (*n* = 73)		Normal weight (*n* = 24)		Overweight (*n* = 32)		Individuals with obesity (*n* = 17)	
	Men (*n* = 35)	Women (*n* = 38)	Men (*n* = 9)	Women (*n* = 15)	Men (*n* = 13)	Women (*n* = 19)	Men (*n* = 13)	Women (*n* = 4)
Age (years)	54.4 ± 5.3	52.9 ± 5.1	55.0 ± 5.4	53.1 ± 4.6	54.4 ± 5.8	53.1 ± 5.3	53.9 ± 4.9	51.8 ± 6.9
Weight (kg)	86.36 ± 11.05	66.36 ± 10.04	73.03 ± 5.68	58.94 ± 5.55	86.19 ± 5.98	68.40 ± 6.61	95.76 ± 7.73	84.46 ± 9.91
Height (m)	175.8 ± 6.5	160.9 ± 6.0	178.2 ± 5.0	162.2 ± 4.9	177.7 ± 6.6	159.8 ± 6.6	172.3 ± 6.2	161.6 ± 7.3
Fat mass (%)	34.59 ± 7.89	45.51 ± 7.51	28.35 ± 5.53	40.02 ± 4.46	33.03 ± 6.14	49.34 ± 7.62	40.46 ± 7.00	47.91 ± 2.03
Fat-free mass (kg)	56.04 ± 6.88	36.01 ± 6.54	52.28 ± 5.36	35.32 ± 4.02	57.77 ± 6.8.0	34.85 ± 7.20	56.92 ± 7.35	44.11 ± 6.47
Lean mass (kg)	53.41 ± 6.71	34.08 ± 6.37	49.70 ± 5.15	33.42 ± 3.97	55.13 ± 6.60	32.94 ± 7.03	54.27 ± 7.22	41.97 ± 6.08
REE (Kcal/day)	1796 ± 196	1291 ± 175	1763 ± 130	1238 ± 190	1806 ± 258	1291 ± 140	1808 ± 173	1495 ± 151

Data are expressed as mean ± standard deviation. Abbreviations: REE, resting energy expenditure.

## Supplementary Materials

The following are available online at http://www.mdpi.com/2072-6643/10/11/1635/s1, File S1: Excel sheet that automatically estimates the REE and the total energy expenditure using 33 equations considering anthropometric and/or body composition parameters (if available), Table S1: Resting energy expenditure predictive equations, Table S2: Validity of resting energy expenditure (REE) predictive equations in normal-weight, overweight, and obese individuals.
