# Supplementary material for: Accuracy and Validity of Resting Energy Expenditure Predictive Equations in Middle-Aged Adults
Source: Nutrients. 2018 Nov 2;10(11):1635. doi: 10.3390/nu10111635 (PMC6266118; doi:10.3390/nu10111635)
Supplement: Supplementary file 1 [file nutrients-10-01635-s001.zip › nutrients-366954-SI/Supplementary files/Table S1.docx]

**Table S1**: Resting energy expenditure predictive equations.

| **Reference** | **Participants** | **Statistics and cross-validation** | **REE predictive equations** |
| --- | --- | --- | --- |
| Harris & Benedict  (1919) | N=239 (136M; 103F), 21-70 y, 25-124.9 kg, 150-200 cm | M: r = 0.86, CL =211  F: r = 0.77, CL = 212 | M: WT*13.7516+HTCM*5.0033–AGE*6.755+66.473  F: WT*9.5634+HTCM*1.8496-AGE*4.6756+655.0955 |
| Roza et al.  (1984) | N=337 (168M; 169F), 21-70 years, 25-124.9 kg, 150-200 cm | M: r = 0.86, CL = 213  F: r = 0.83, CL = 201 | M: 13.397*WT+4.799*HTCM–5.677*AGE+88.362  F: 9.247*WT+3.098*HTCM–4.33*AGE+477.593 |
| Bernstein et al.  (1983) | N=202 (48 M; 154 F), 28-52 y, 60-204 kg, 157-182 cm, BMI>30 | M: R^2^=0.449  F: R^2^=0.657  R^2^=0.485 | M: 11.02*WT+10.23*HTCM-5.8*AGE-1032  F: 7.48*WT-0.42*HTCM-3*AGE+844  19.02*FFM+3.72*FM-1.55*AGE+236.7 |
| Owen et al.  (1986) | N=104 (60 M; 44 F), 18–82 y, 60-171 kg (M) 43-153 kg (F), BMI 18–50 | M: R^2^=0.71  F: R^2^=0.74  M: R^2^=0.74  F: R^2^=0.71 | M: WT*10.2+879  F: WT*7.18+795  M: 22.3*FFM+290  F: 19.7*FFM+334 |
| Mifflin et al.  (1990) | N=498 (251 M; 248 F), N=264 normal weight (129 M; 135 F), N=234 individuals with obesity (122 M; 112 F), 19–78 y, BMI 17–42 | R^2^= 0.71  R^2^= 0.64 | 9.99*WT+6.25*HTCM-4.92*AGE+166*SEX–161  19.7*FFM+413 |
| Livingston et al.  (2005) | N=655 (299 M; 356 F), 18–95 y, 33–  278 kg | M: R^2^= 0.77  F: R^2^= 0.71 | M: 293*WT^0.4330^– 5.92*AGE  F: 248*WT^0.4356^–5.09*AGE |
|  |  |  |  |
| Schofield et al.  (1985) | N=7,173, N=4,814>18 y, BMI 21–24  N= 3,388 Italians (47%), N=615 tropical residents, N= 322 Indian  114 published studies, N=7,173 subjects (11,000 values, includes group mean values); most European and North American subjects | M: 30-60y: r=0.60  >60y: r=0.74  F: 30-60y: r=0.68  >60y: r=0.73  M: 30-60y: r=0.60  >60y: r=0.74  F: 30-60y: r=0.68  >60y: r=0.73 | M: 30-60y: 11,472*WT–873.1  >60y: 11.711*WT+587.7  F: 30-60y: 8,126*WT+845.6  >60y: 9.082*WT+658.5  M: 30-60y: 0.048*WT– 0.011*HTM+3.67  >60y: 0.038*WT+4.068*HTM+3.491  F: 30-60y: 0.034*WT+0.006*HTM+3.53  >60y: 0.033*WT+1.917*HTM+0.074 |
| FAO  (1985) | Equation based on Schofield et al (1985);  database extended to 11,000 subjects | M: 30-60y; r=0.6  >60y: r=0.79  F: 30-60y: r= 0.7  >60y: R=0.74  M: 30-60y: r=0.6  >60y: 0.84  F: 30-60y: r=0.7  >60y: r=0.82 | M: 30-60y: 11.6*WT+879  >60y: 13.5*WT+487  F: 30-60y: 8.7*WT+829  >60y: 10.5*WT+596  M: 30-60y: 11.3*Weight–16*Height+901  >60y: 8.8*WT+1128*HTM–1071  F: 30-60y: 8.7*WT–25*HTM+865  >60y: 9.2*WT+637*HTM–302 |
| Henry et al.  (2005) | N=10,552 (5794 M; 4702 F) | M: 30-60y: r=0.742  >60y: r=0.776  F: 30-60y: r=0.690  >60y: 0.786  M: 30-60y: r=0.756  >60y: r=0.789  F: 30-60y: r=0.713  >60y: 0.805 | M: 30-60y: 0.0592*WT+2.48  >60y: 0.0563*WT+2.15  F: 30-60y: 0.0407*WT+2.9  >60y: 0.0424*WT+2.38  M: 30-60y: 0.0476*WT+2.26*HTM–0.574  >60y: 0.0478*WT+2.26*HTM–1.07  F: 30-60y: 0.0342*WT+2.1*HTM–0.0486  >60y: 0.0356*WT+1.76*HTM+0.0448 |
| Muller et al.  (2004) | N=2,528 (1027 M; 1501 F), 5–80 y, BMI >25 | r=0.83 | 0.047*WT– 0.01452*AGE+1.009*SEX+3.21 |
|  |  | r=0.79 | BMI 25–30: 0.04507*WT-0.01553*AGE+1.006*SEX+3.407 |
|  |  | r=0.84 | BMI >30: 0.05*WT-0.01586*AGE+1.103*SEX+2.924 |
|  |  | r=0.83 | 0.05192*FFM+0.04036*FM+0.869*SEX-0.01181*AGE+2.992 |
|  |  | r=0.79 | BMI 25–30: 0.03776*FFM+0.03013*FM+0.93*SEX-0.01196*AGE+3.928 |
|  |  | r=0.84 | BMI >30: 0.05685*FFM+0.04022*FM+0.808*SEX-0.01402*AGE+2.818 |
| Korth et al.  (2007) | N=104 (50 M; 54 F), 21–68 y, BMI 18-41 | *r* = 0.84, *R*^2^= 0.71, SE = 788 | 41.5*WT+35.0*HTCM+1107.4*SEX-19.1*AGE-1731.2 |
|  |  | *r* = 0.86, *R*^2^= 0.74, SE = 732 | 108.1*FFM+1231 |
| De Lorenzo et al.  (2001) | N=320 (127 M; 193 F), 18–59 y, BMI 17–40 | F: R^2^=0.597, SE=650 | M: 53.284*WT+20.957*HTCM–23.859*AGE+487 |
|  |  | M: R^2^=0.597, SE=581 | F: 46.322*WT+15.744*HTCM–16.66*AGE+944 |
| Lazzer et al.  (2007) | N= 346 (164 M; 182 F), 20–65 y, mean BMI 45 (50% FM) | M: R^2^=0.68, SE=1.14 | M: 0.048*WT+4.655*HTM-0.020*AGE-3.605 |
|  |  | F: R^2^=0.66, SE=0.56 | F: 0.042*WT+3.619*HTM-2.678 |
| Johnstone et al.  (2006) | N=150 (43 M; 107 F), 21–64 y, BMI 17–49 | R^2^=0.774 | 90.2*FFM+31.6*FM-12.2*AGE+1613 |
| Weijs et al.  (2010) | N=536 F, >19 y, BMI >28 | R^2^ = 0.69, SEE = 204 | WT*14.038+HTCM*4.498+SEX*137.566−AGE*0.977−221.631 |
| Frankenfield  (2015) | N=337, >18 y | R^2^=0.84 | BMI≥30: WT*10−AGE*5+SEX*274+865  BMI<30: WT*11-AGE*6+SEX*230+838  BMI≥30: WT*10+HTCM*3−AGE*5+SEX*244+440  BMI<30: WT*10+HTCM*3−AGE*5+SEX*207+454 |
| De la Cruz et al.  (2014) | N=134 (67 M; 67 F), 19-65 y | R^2^=0.68 | 1376,4–308*SEX***+11,1*WT–8*AGE |
| Willis et al.  (2015) | N=159, 18-30 y, BMI mean 30.7 | R^2^=0.77 | 11.2*WT−7.2*AGE+237.6*SEX+780.3 |
| Cunningham  (1980) | N=223 (120M; 103F) | R^2^=0.7 | 500 + 22*Lean Body Mass |
| Huang  (2004) | N=1088 (279M; 759F) | R^2^=0.737  R^2^=0.723 | 10.158*WT+3.933*HTCM–1.44*Age+273.821*Sex+60.655  14.118*FFM+9.367*FM–1.515*Age + 220.863*Sex+521.995 |
| De Luis et al.  (2006) | N=200 (60 M; 140 F), >20 y, BMI>30 | M: R^2^=0.70  F: R^2^=0.70 | M: 58.6+(6.1*WT)+(1023.7*HTM)–(9.5*AGE)  F: 1272.5+(9.8*WT)–(61.6*HTM)–(8.2*AGE) |

Abbreviations: M, male; F, female; y, years of age; kg, kilograms; cm, centimeters; BMI, body mass index; WT, weight; HTCM, height in centimeters; FFM, fat free mass; FM, fat mass; HTM, height in meters; r and r^2^ values of the correlation between each predictive equation and the indirect calorimetry measurement in the original paper; CL, confident limit. ***Female*1, male*0.
