# Supplementary material for: Accuracy and Validity of Resting Energy Expenditure Predictive Equations in Middle-Aged Adults
Source: Nutrients. 2018 Nov 2;10(11):1635. doi: 10.3390/nu10111635 (PMC6266118; doi:10.3390/nu10111635)
Supplement: Supplementary file 1 [file nutrients-10-01635-s001.zip › nutrients-366954-SI/Supplementary files/Table S2.docx]

**Table S2A:** Validity of resting energy expenditure (REE) predictive equations in normal-weight adults.

| **REE predictive equation** | **N** | **^1^REE**  **(Kcal/day)** | **P value ANCOVA^2^** | **Mean BIAS^3^ (Kcal/day)** | **Lower limit of agreement (Kcal/day)** | **Higher limit of agreement (Kcal/day)** | **Mean absolute differences^4^ (Kcal/day)** | **Percentage of accurate predictions (10%)^5^** | **Percentage of under predictions (10%)^6^** | **Percentage of over predictions (10%)^7^** | **Percentage of accurate predictions (5%)^8^** | **Percentage of under predictions (5%)^9^** | **Percentage of over predictions (5%)^10^** |
| --- | --- | --- | --- | --- | --- | --- | --- | --- | --- | --- | --- | --- | --- |
| Harris & Benedict | 24 | 1391 ± 174 | 0.660 | 45 | -405 | 586 | 146 ± 143 | 50.0 | 20.8 | 29.2 | 37.5 | 20.8 | 41.7 |
| Roza | 24 | 1413 ± 171 | 0.668 | 23 | -424 | 565 | 142 ± 143 | 58.3 | 20.8 | 20.8 | 41.7 | 25.0 | 33.3 |
| Owen_a | 24 | 1370 ± 206 | 0.764 | 65 | -376 | 508 | 143 ± 124 | 62.5 | 12.5 | 25.0 | 33.3 | 20.8 | 45.8 |
| Owen_b | 24 | 1192 ± 230 | 0.486 | 244 | -166 | 671 | 262 ± 162 | 20.8 | 4.2 | 75.0 | 16.7 | 4.2 | 79.2 |
| Mifflin_a | 24 | 1329 ± 210 | 0.951 | 106 | -287 | 566 | 164 ± 126 | 45.8 | 8.3 | 45.8 | 16.7 | 20.8 | 62.5 |
| Mifflin_b | 24 | 1234 ± 187 | 0.728 | 201 | -242 | 671 | 237 ± 156 | 20.8 | 8.3 | 70.8 | 8.3 | 12.5 | 79.2 |
| Livingston | 24 | 1327 ± 188 | 0.725 | 108 | -332 | 582 | 168 ± 133 | 50.0 | 8.3 | 41.7 | 20.8 | 16.7 | 62.5 |
| Schofield | 24 | 1217 ± 337 | 0.521 | 219 | -125 | 548 | 232 ± 158 | 29.2 | 4.2 | 66.7 | 16.7 | 4.2 | 79.2 |
| Schofield_ht | 24 | 1453 ± 172 | 0.745 | -17 | -484 | 363 | 129 ± 132 | 66.7 | 20.8 | 12.5 | 45.8 | 25.0 | 29.2 |
| FAO | 24 | 1460 ± 167 | 0.458 | -24 | -499 | 347 | 138 ± 136 | 58.3 | 20.8 | 20.8 | 41.7 | 29.2 | 29.2 |
| FAO_ht | 24 | 1460 ± 164 | 0.785 | -24 | -496 | 373 | 131 ± 138 | 66.7 | 20.8 | 12.5 | 50.0 | 25.0 | 25.0 |
| Henry | 24 | 1433 ± 160 | 0.818 | 2 | -455 | 551 | 143 ± 144 | 58.3 | 20.8 | 20.8 | 41.7 | 25.0 | 33.3 |
| Henry_ht | 24 | 1508 ± 287 | 0.463 | -72 | -431 | 237 | 143 ± 117 | 58.3 | 33.3 | 8.3 | 33.3 | 45.8 | 20.8 |
| Muller_a | 24 | 1392 ± 204 | 0.846 | 44 | -382 | 498 | 133 ± 125 | 58.3 | 16.7 | 25.0 | 41.7 | 20.8 | 37.5 |
| Muller_b | 24 | 1375 ± 200 | 0.918 | 61 | -368 | 517 | 140 ± 126 | 62.5 | 12.5 | 25.0 | 33.3 | 20.8 | 45.8 |
| Korth_a | 24 | 1483 ± 274 | 0.989 | -47 | -379 | 339 | 141 ± 101 | 62.5 | 29.2 | 8.3 | 25.0 | 45.8 | 29.2 |
| Korth_b | 24 | 1370 ± 245 | 0.743 | 65 | -321 | 500 | 165 ± 117 | 50.0 | 12.5 | 37.5 | 16.7 | 29.2 | 54.2 |
| De Lorenzo | 24 | 1407 ± 189 | 0.738 | 29 | -393 | 543 | 139 ± 133 | 54.2 | 20.8 | 25.0 | 37.5 | 25.0 | 37.5 |
| Johnstone_b | 24 | 1297 ± 193 | 0.973 | 139 | -284 | 656 | 189 ± 152 | 45.8 | 8.3 | 45.8 | 20.8 | 12.5 | 66.7 |
| Weijs | 24 | 1435 ± 220 | 0.855 | 0 | -382 | 502 | 132 ± 124 | 62.5 | 20.8 | 16.7 | 41.7 | 29.2 | 29.2 |
| Frankenfield | 24 | 1308 ± 196 | 0.725 | 128 | -306 | 586 | 176 ± 134 | 41.7 | 8.3 | 50.0 | 16.7 | 12.5 | 70.8 |
| Frankenfield_ht | 24 | 1379 ± 225 | 0.855 | 57 | -341 | 479 | 137 ± 115 | 62.5 | 8.3 | 29.2 | 33.3 | 20.8 | 45.8 |
| De la Cruz | 24 | 1603 ± 240 | 0.076 | -167 | -748 | 186 | 229 ± 181 | 45.8 | 45.8 | 8.3 | 16.7 | 66.7 | 16.7 |
| Cunningham | 24 | 1370 ± 201 | 0.899 | 66 | -357 | 524 | 160 ± 129 | 62.5 | 12.5 | 25.0 | 20.8 | 20.8 | 58.3 |
| Huang_a | 24 | 1400 ± 245 | 0.717 | 36 | -341 | 446 | 133 ± 107 | 62.5 | 12.5 | 25.0 | 29.2 | 25.0 | 45.8 |
| Huang_b | 24 | 1323 ± 223 | 0.761 | 113 | -295 | 550 | 161 ± 129 | 41.7 | 8.3 | 50.0 | 29.2 | 12.5 | 58.3 |

^1^REE obtained by predictive equations (Mean±SD); ^2^P value of the main effect of ANCOVA comparing measured and predicted REE adjusting for age; ^3^Mean error between measured value and predictive equation (measured – predicted); ^4^Mean of absolute differences between measured and predictive value (Mean±SD); ^5^Percentage of subjects predicted by this predictive equation within ±10% of the measured value; ^6^Percentage of subjects predicted by this predictive equation <10% of the measured value; ^7^Percentage of subjects predicted by this predictive equation >10% of the measured value; ^8^Percentage of subjects predicted by this predictive equation within ±10% of the measured value; ^9^Percentage of subjects predicted by this predictive equation <10% of the measured value; ^10^Percentage of subjects predicted by this predictive equation >10% of the measured value. *P<0.05, **P<0.01, ***P<0.001, ANCOVA test. “_a” refers to predictive equations which required only anthropometric parameters to calculate REE, “_b” refers to predictive equations which required body composition parameters to calculate REE, and “_ht” refers to predictive equations which are proposed by the same author and include height.

**Table S2B:** Validity of resting energy expenditure (REE) predictive equations in overweight adults.

| **REE predictive equation** | **N** | **REE^1^**  **(Kcal/day)** | **P value ANCOVA^2^** | **Mean BIAS^3^ (Kcal/day)** | **Lower limit of agreement (Kcal/day)** | **Higher limit of agreement (Kcal/day)** | **Mean absolute differences^4^ (Kcal/day)** | **Percentage of accurate predictions (10%)^5^** | **Percentage of under predictions (10%)^6^** | **Percentage of over predictions (10%)^7^** | **Percentage of accurate predictions (5%)^8^** | **Percentage of under predictions (5%)^9^** | **Percentage of over predictions (5%)^10^** |
| --- | --- | --- | --- | --- | --- | --- | --- | --- | --- | --- | --- | --- | --- |
| Harris & Benedict | 32 | 1526 ± 231 | 0,285 | -25 | -340 | 456 | 125 ± 98 | 71.9 | 18.8 | 9.4 | 40.6 | 40.6 | 18.8 |
| Roza | 32 | 1543 ± 229 | 0,282 | -42 | -362 | 447 | 128 ± 102 | 65.6 | 25.0 | 9.4 | 31.3 | 53.1 | 15.6 |
| Owen_a | 32 | 1478 ± 241 | 0,008 | 23 | -360 | 516 | 120 ± 121 | 71.9 | 12.5 | 15.6 | 46.9 | 21.9 | 31.3 |
| Owen_b | 32 | 1249 ± 312 | 0,042 | 251 | -117 | 549 | 259 ± 131 | 12.5 | 0.0 | 87.5 | 6.3 | 3.1 | 90.6 |
| Mifflin_a | 32 | 1442 ± 250 | 0,189 | 58 | -299 | 538 | 123 ± 110 | 78.1 | 3.1 | 18.8 | 31.3 | 18.8 | 50.0 |
| Mifflin_b | 32 | 1283 ± 263 | 0,071 | 216 | -97 | 598 | 224 ± 139 | 25.0 | 0.0 | 75.0 | 9.4 | 3.1 | 87.5 |
| Livingston | 32 | 1455 ± 216 | 0,142 | 46 | -249 | 562 | 117 ± 122 | 75.0 | 9.4 | 15.6 | 46.9 | 18.8 | 34.4 |
| Schofield | 32 | 1241 ± 336 | 0,003 | 260 | -147 | 606 | 282 ± 167 | 25.0 | 0.0 | 75.0 | 18.8 | 6.3 | 75.0 |
| Schofield_ht | 32 | 1444 ± 194 | 0,228 | 57 | -222 | 607 | 131 ± 137 | 71.9 | 6.3 | 21.9 | 43.8 | 18.8 | 37.5 |
| FAO | 32 | 1449 ± 188 | 0,544 | 51 | -232 | 590 | 135 ± 140 | 71.9 | 6.3 | 21.9 | 37.5 | 31.3 | 31.3 |
| FAO_ht | 32 | 1451 ± 185 | 0,193 | 49 | -249 | 618 | 133 ± 139 | 71.9 | 6.3 | 21.9 | 34.4 | 31.3 | 34.4 |
| Henry | 32 | 1586 ± 198 | 0,282 | -86 | -351 | 447 | 160 ± 107 | 50.0 | 40.6 | 9.4 | 25.0 | 62.5 | 12.5 |
| Henry_ht | 32 | 1615 ± 339 | 0,892 | -115 | -474 | 219 | 150 ± 132 | 59.4 | 37.5 | 3.1 | 37.5 | 56.3 | 6.3 |
| Muller_a | 32 | 1527 ± 226 | 0,117 | -26 | -351 | 481 | 124 ± 105 | 68.8 | 18.8 | 12.5 | 40.6 | 43.8 | 15.6 |
| Muller_b | 32 | 1500 ± 204 | 0,092 | 1 | -305 | 532 | 123 ± 116 | 68.8 | 15.6 | 15.6 | 40.6 | 34.4 | 25.0 |
| Korth_a | 32 | 1596 ± 316 | 0,336 | -95 | -545 | 300 | 130 ± 129 | 62.5 | 34.4 | 3.1 | 43.8 | 50.0 | 6.3 |
| Korth_b | 32 | 1434 ± 345 | 0,423 | 66 | -313 | 422 | 132 ± 107 | 65.6 | 3.1 | 31.3 | 28.1 | 18.8 | 53.1 |
| De Lorenzo | 32 | 1543 ± 236 | 0,319 | -42 | -369 | 444 | 124 ± 104 | 59.4 | 31.3 | 9.4 | 40.6 | 43.8 | 15.6 |
| Johnstone_b | 32 | 1418 ± 267 | 0,36 | 83 | -222 | 473 | 120 ± 112 | 65.6 | 3.1 | 31.3 | 46.9 | 9.4 | 43.8 |
| Weijs | 32 | 1595 ± 260 | 0,158 | -95 | -484 | 374 | 144 ± 115 | 56.3 | 37.5 | 6.3 | 37.5 | 53.1 | 9.4 |
| Frankenfield | 32 | 1442 ± 225 | 0,921 | 59 | -242 | 564 | 118 ± 122 | 71.9 | 6.3 | 21.9 | 40.6 | 18.8 | 40.6 |
| Frankenfield_ht | 32 | 1498 ± 257 | 0,823 | 2 | -358 | 470 | 111 ± 103 | 75.0 | 12.5 | 12.5 | 34.4 | 37.5 | 28.1 |
| De la Cruz | 32 | 1520 ± 359 | 0,599 | -19 | -600 | 754 | 258 ± 219 | 46.9 | 34.4 | 18.8 | 18.8 | 53.1 | 28.1 |
| Cunningham | 32 | 1423 ± 285 | 0,731 | 78 | -259 | 417 | 121 ± 114 | 65.6 | 3.1 | 31.3 | 46.9 | 9.4 | 43.8 |
| Huang_a | 32 | 1520 ± 276 | 0,529 | -20 | -438 | 430 | 115 ± 108 | 75.0 | 15.6 | 9.4 | 43.8 | 31.3 | 25.0 |
| Huang_b | 32 | 1449 ± 262 | 0,464 | 52 | -317 | 491 | 114 ± 109 | 71.9 | 3.1 | 25.0 | 43.8 | 15.6 | 40.6 |

^1^REE obtained by predictive equations (Mean±SD); ^2^P value of the main effect of ANCOVA comparing measured and predicted REE adjusting for age; ^3^Mean error between measured value and predictive equation (measured – predicted); ^4^Mean of absolute differences between measured and predictive value (Mean±SD); ^5^Percentage of subjects predicted by this predictive equation within ±10% of the measured value; ^6^Percentage of subjects predicted by this predictive equation <10% of the measured value; ^7^Percentage of subjects predicted by this predictive equation >10% of the measured value; ^8^Percentage of subjects predicted by this predictive equation within ±10% of the measured value; ^9^Percentage of subjects predicted by this predictive equation <10% of the measured value; ^10^Percentage of subjects predicted by this predictive equation >10% of the measured value. *P<0.05, **P<0.01, ***P<0.001, ANCOVA test. “_a” refers to predictive equations which required only anthropometric parameters to calculate REE, “_b” refers to predictive equations which required body composition parameters to calculate REE, and “_ht” refers to predictive equations which are proposed by the same author and include height.

**Table S2C:** Validity of resting energy expenditure (REE) predictive equations in individuals with obesity.

| **REE predictive equation** | **N** | **REE^1^**  **(Kcal/day)** | **P value ANCOVA^2^** | **Mean BIAS^3^ (Kcal/day)** | **Lower limit of agreement (Kcal/day)** | **Higher limit of agreement (Kcal/day)** | **Mean absolute differences^4^ (Kcal/day)** | **Percentage of accurate predictions (10%)^5^** | **Percentage of under predictions (10%)^6^** | **Percentage of over predictions (10%)^7^** | **Percentage of accurate predictions (5%)^8^** | **Percentage of under predictions (5%)^9^** | **Percentage of over predictions (5%)^10^** |
| --- | --- | --- | --- | --- | --- | --- | --- | --- | --- | --- | --- | --- | --- |
| Harris & Benedict | 17 | 1796 ± 216 | 0.059 | -61 | -748 | 177 | 153 ± 177 | 76.5 | 17.6 | 5.9 | 35.3 | 35.3 | 29.4 |
| Roza | 17 | 1808 ± 211 | 0.062 | -73 | -747 | 151 | 149 ± 179 | 82.4 | 17.6 | 0.0 | 47.1 | 41.2 | 11.8 |
| Bernstein_a | 17 | 1422 ± 175 | 0.196 | 313 | -346 | 594 | 354 ± 166 | 11.8 | 5.9 | 82.4 | 5.9 | 5.9 | 88.2 |
| Bernstein_b | 17 | 1325 ± 165 | 0.574 | 410 | -4 | 694 | 410 ± 202 | 5.9 | 0.0 | 94.1 | 5.9 | 0.0 | 94.1 |
| Owen_a | 17 | 1749 ± 212 | 0.513 | -14 | -591 | 245 | 132 ± 138 | 76.5 | 11.8 | 11.8 | 52.9 | 23.5 | 23.5 |
| Owen_b | 17 | 1477 ± 217 | 0.688 | 258 | -111 | 598 | 280 ± 185 | 29.4 | 0.0 | 70.6 | 17.6 | 5.9 | 76.5 |
| Mifflin_a | 17 | 1695 ± 198 | 0.076 | 40 | -587 | 271 | 164 ± 133 | 64.7 | 5.9 | 29.4 | 23.5 | 17.6 | 58.8 |
| Mifflin_b | 17 | 1475 ± 176 | 0.613 | 260 | -89 | 593 | 273 ± 187 | 29.4 | 0.0 | 70.6 | 5.9 | 5.9 | 88.2 |
| Livingston | 17 | 1710 ± 178 | 0.154 | 25 | -535 | 247 | 145 ± 119 | 70.6 | 5.9 | 23.5 | 23.5 | 23.5 | 52.9 |
| Schofield | 17 | 1462 ± 281 | 0.983 | 273 | -305 | 669 | 309 ± 200 | 23.5 | 5.9 | 70.6 | 17.6 | 5.9 | 76.5 |
| Schofield_ht | 17 | 1528 ± 182 | 0.17 | 206 | -304 | 504 | 242 ± 154 | 29.4 | 5.9 | 64.7 | 17.6 | 5.9 | 76.5 |
| FAO | 17 | 1541 ± 186 | 0.137 | 194 | -321 | 501 | 232 ± 162 | 29.4 | 5.9 | 64.7 | 23.5 | 5.9 | 70.6 |
| FAO_ht | 17 | 1526 ± 171 | 0.18 | 209 | -292 | 503 | 243 ± 152 | 29.4 | 5.9 | 64.7 | 17.6 | 5.9 | 76.5 |
| Henry | 17 | 1866 ± 186 | 0.05 | -131 | -743 | 97 | 161 ± 181 | 64.7 | 35.3 | 0.0 | 47.1 | 47.1 | 5.9 |
| Henry_ht | 17 | 1953 ± 339 | 0.053 | -218 | -963 | 210 | 283 ± 232 | 47.1 | 47.1 | 5.9 | 5.9 | 70.6 | 23.5 |
| Muller_a | 17 | 1791 ± 194 | 0.121 | -56 | -655 | 164 | 137 ± 149 | 82.4 | 17.6 | 0.0 | 41.2 | 35.3 | 23.5 |
| Muller_b | 17 | 1750 ± 185 | 0.143 | -16 | -566 | 217 | 145 ± 123 | 82.4 | 11.8 | 5.9 | 29.4 | 29.4 | 41.2 |
| Korth_a | 17 | 1888 ± 246 | 0.06 | -153 | -826 | 59 | 174 ± 210 | 70.6 | 29.4 | 0.0 | 52.9 | 47.1 | 0.0 |
| Korth_b | 17 | 1686 ± 230 | 0.34 | 49 | -311 | 435 | 187 ± 139 | 52.9 | 11.8 | 35.3 | 41.2 | 23.5 | 35.3 |
| De Lorenzo | 17 | 1813 ± 203 | 0.047 | -78 | -737 | 153 | 149 ± 177 | 76.5 | 23.5 | 0.0 | 41.2 | 41.2 | 17.6 |
| Lazzer | 17 | 1827 ± 205 | 0.038 | -92 | -779 | 178 | 168 ± 186 | 64.7 | 29.4 | 5.9 | 41.2 | 41.2 | 17.6 |
| Johnstone_b | 17 | 1687 ± 186 | 0.112 | 48 | -438 | 327 | 179 ± 120 | 64.7 | 11.8 | 23.5 | 17.6 | 29.4 | 52.9 |
| Weijs | 17 | 1902 ± 205 | 0.055 | -167 | -826 | 63 | 178 ± 206 | 64.7 | 35.3 | 0.0 | 47.1 | 52.9 | 0.0 |
| Frankenfield | 17 | 1738 ± 192 | 0.921 | -4 | -579 | 213 | 138 ± 129 | 76.5 | 5.9 | 17.6 | 35.3 | 23.5 | 41.2 |
| Frankenfield_ht | 17 | 1800 ± 202 | 0.823 | -65 | -671 | 153 | 139 ± 157 | 82.4 | 17.6 | 0.0 | 41.2 | 35.3 | 23.5 |
| De la Cruz | 17 | 1721 ± 222 | 0.599 | 14 | -633 | 535 | 162 ± 173 | 76.5 | 5.9 | 17.6 | 35.3 | 29.4 | 35.3 |
| Cunningham | 17 | 1630 ± 190 | 0.731 | 104 | -239 | 460 | 181 ± 148 | 52.9 | 11.8 | 35.3 | 35.3 | 17.6 | 47.1 |
| Huang_a | 17 | 1807 ± 216 | 0.529 | -72 | -686 | 137 | 135 ± 165 | 76.5 | 23.5 | 0.0 | 52.9 | 29.4 | 17.6 |
| Huang_b | 17 | 1738 ± 196 | 0.464 | -3 | -552 | 222 | 143 ± 123 | 76.5 | 11.8 | 11.8 | 35.3 | 23.5 | 41.2 |
| De Luis | 17 | 1820 ± 195 | 0.333 | -85 | -705 | 150 | 158 ± 173 | 76.5 | 23.5 | 0.0 | 41.2 | 41.2 | 17.6 |

^1^REE obtained by predictive equations (Mean±SD); ^2^P value of the main effect of ANCOVA comparing measured and predicted REE adjusting for age; ^3^Mean error between measured value and predictive equation (measured – predicted); ^4^Mean of absolute differences between measured and predictive value (Mean±SD); ^5^Percentage of subjects predicted by this predictive equation within ±10% of the measured value; ^6^Percentage of subjects predicted by this predictive equation <10% of the measured value; ^7^Percentage of subjects predicted by this predictive equation >10% of the measured value; ^8^Percentage of subjects predicted by this predictive equation within ±10% of the measured value; ^9^Percentage of subjects predicted by this predictive equation <10% of the measured value; ^10^Percentage of subjects predicted by this predictive equation >10% of the measured value. *P<0.05, **P<0.01, ***P<0.001, ANCOVA test. “_a” refers to predictive equations which required only anthropometric parameters to calculate REE, “_b” refers to predictive equations which required body composition parameters to calculate REE, and “_ht” refers to predictive equations which are proposed by the same author and include height.
